# Impact of Preoperative Teriparatide Use on Proximal Junctional Kyphosis Prevention in Osteopenic/Osteoporotic Patients Undergoing Adult Spinal Deformity Surgery: A Propensity Score-Matched Study

**DOI:** 10.3390/jcm15145682

**Published:** 2026-07-20

**Authors:** Jun-Seok Oh, Jin-Sung Park, Dong-Ho Kang, Seungjae Park, Tae Soo Shin, Se-Jun Park

**Affiliations:** Department of Orthopedic Surgery, Samsung Medical Center, Sungkyunkwan University School of Medicine, Seoul 06351, Republic of Korea

**Keywords:** adult spinal deformity, proximal junctional kyphosis, teriparatide, osteoporosis, propensity score matching

## Abstract

**Background/Objectives**: Poor bone quality significantly increases the risk of mechanical complications after adult spinal deformity (ASD) surgery, including proximal junctional kyphosis (PJK) and proximal junctional failure (PJF). Although teriparatide enhances bone quality, its effectiveness in preventing junctional complications after extensive correction remains unclear. This study investigated whether preoperative teriparatide administration reduces the incidence of PJK and PJF in patients with compromised bone quality undergoing ASD surgery. **Methods**: This retrospective cohort study reviewed 292 patients (T-score < −1.0) who underwent ASD surgery between 2015 and 2023. Patients were divided into teriparatide (*n* = 59) and non-teriparatide (*n* = 233) groups. Propensity score matching (1:2 ratio) was performed using six covariates: age, preoperative pelvic incidence minus lumbar lordosis, preoperative T1 pelvic angle, upper instrumented vertebra (UIV) cementing, UIV screw angle, and fusion length, yielding 153 matched patients (52 teriparatide, 101 non-teriparatide). **Results**: After matching, all baseline characteristics were statistically similar, with no significant differences. The incidence of PJK and PJF did not differ significantly between the teriparatide and non-teriparatide groups (44.2% vs. 52.5%, *p* = 0.426; 9.6% vs. 9.9%, *p* = 1.000), nor did PJK subtype distribution (bony vs. soft-tissue). Multivariate analysis identified older age (odds ratio [OR] = 1.058), higher American Society of Anesthesiologists score (OR = 2.603, *p* = 0.002), UIV at the thoracolumbar junction (OR = 2.786), and greater postoperative thoracic kyphosis (OR = 1.044) as independent risk factors for PJK. Teriparatide use was not an independent predictor (OR = 0.872). **Conclusions**: Preoperative teriparatide did not significantly reduce the incidence of PJK or PJF after long-segment fusion for ASD. These findings suggest that improving bone quality alone is insufficient to prevent junctional complications, which are driven by complex biomechanical and patient-related factors inherent to extensive deformity correction.

## 1. Introduction

Proximal junctional kyphosis (PJK) is one of the most common mechanical complications following corrective surgery for adult spinal deformity (ASD). Poor bone quality is a critical determinant of surgical outcomes in spinal fusion surgery, particularly in multilevel instrumented fusion for ASD, and significantly increases the risk of mechanical complications, including instrumentation failure and PJK [1,2,3,4,5,6].

Osteoporosis and osteopenia are major contributors to compromised bone quality, and their prevalence is steadily increasing, posing a growing clinical challenge in ASD surgery [7,8]. A recent systematic review reported that 34.2% and 43.5% of patients older than 50 years undergoing spinal surgery had osteoporosis and osteopenia, respectively, indicating that up to 78.7% of these patients have compromised bone quality [7].

Teriparatide, a recombinant parathyroid hormone analog with anabolic bone-forming properties, has shown beneficial effects on bone quality and fusion-related outcomes after spinal fusion surgery [9,10,11,12,13]. Recent studies suggest that teriparatide may also improve outcomes in long-segment fusion for ASD [14,15]. Although perioperative teriparatide may theoretically reduce PJK by improving bone quality around the proximal junction, only a limited number of studies have specifically evaluated its preventive effect on proximal junctional complications [14,15,16,17].

The present study aims to investigate whether preoperative teriparatide use prevents the development of PJK in patients with compromised bone quality, including osteopenia or osteoporosis, undergoing corrective surgery for ASD. We analyzed PJK and proximal junctional failure (PJF) rates between teriparatide-treated and untreated patients, evaluated PJK subtypes, and identified independent risk factors for PJK development.

## 2. Materials and Methods

**Study Design:** This retrospective cohort study analyzed data from a prospectively collected ASD database at a single tertiary referral center. The institutional review board (IRB No. 2026-06-095) approved the study and waived the requirement for informed consent due to its retrospective nature.

**Study Population:** The study included consecutive patients who underwent corrective surgery for symptomatic ASD between 2015 and 2023. Inclusion criteria were as follows: (1) patients aged ≥50 years; (2) ASD defined by at least one of the following parameters: pelvic incidence minus lumbar lordosis (PI-LL) mismatch ≥ 10°, pelvic tilt (PT) ≥ 25°, and sagittal vertical axis (SVA) ≥ 5 cm; (3) T-score less than −1.0; (4) fusion involving ≥5 vertebrae, including the sacrum or pelvis; and (5) completion of a 2-year follow-up. All surgeries were performed by three orthopedic spine surgeons. Pelvic fixation using conventional iliac screws was routinely performed in primary lumbosacral fusion, while fusion was limited to the sacrum without pelvic fixation in patients with milder deformities and pre-existing lumbosacral solid fusion, either from prior surgery or due to a lumbosacral transitional vertebra. Exclusion criteria included (1) non-degenerative conditions such as infection and tumors; (2) Parkinson’s disease; or (3) revision surgery for reasons unrelated to junctional complications. Based on these eligibility criteria, a total of 292 patients were included in the final analysis, comprising 59 patients in the teriparatide group and 233 patients in the non-teriparatide group.

**Preoperative Use of Teriparatide:** Preoperative use of teriparatide (20 μg/day subcutaneous injection) was indicated for patients deemed to have poor bone quality (i.e., T-score < −1.0) based on preoperative central dual-energy X-ray absorptiometry (DEXA) of the lumbar spine and hip. The decision to initiate teriparatide was made clinically by one of the three treating orthopedic spine surgeons, considering bone quality assessments, overall patient profiles, and surgical risk, rather than being assigned by a predefined study protocol. Patients were classified into the teriparatide group only if they had received teriparatide for at least 3 months preoperatively. This 3-month minimum preoperative duration was established based on previous studies that used similar criteria for defining the teriparatide treatment group [16,18].

**Data Collection:** Demographic variables included sex, age, body mass index (BMI), American Society of Anesthesiologists (ASA) grade, modified frailty index (FI-5) score, T-score, and Hounsfield units at the upper instrumented vertebra (UIV). Bone mineral density was assessed using central DEXA of the lumbar spine and hip, and the lowest T-score among these measured sites was used for analysis. Because all included patients were 50 years of age or older, T-scores rather than Z-scores were used for analysis. Surgical variables included history of prior lumbar fusion, use of lateral lumbar interbody fusion, pelvic fixation, UIV cementing, UIV screw angle, and fusion length. Radiographic parameters included PI-LL, SS, PT, thoracic kyphosis (TK), T1 pelvic angle (TPA), and SVA, measured preoperatively and at immediate postoperative time points. In addition to these postoperative sagittal parameters, several alignment assessment schemes were evaluated, such as Global Alignment and Proportion (GAP) score, Sagittal Age-Adjusted Score (SAAS), and Roussouly classification [19,20,21].

**Outcome measures:** PJK was defined as a proximal junctional angle ≥ 10° and at least 10° greater than the preoperative measurement. PJK was further classified as bony PJK (vertebral fractures at the UIV or UIV + 1, or fixation failure at the UIV) or soft PJK (ligamentous failure without fracture or fixation failure). PJF was defined as PJK cases requiring revision surgery. Additionally, clinical outcomes were evaluated using the Oswestry disability index (ODI), Scoliosis Research Society-22r questionnaire (SRS-22r), and Short Form-36 Physical Component Score (SF-36 PCS) preoperatively and at 2 years postoperatively.

**Propensity Score Matching:** Given that PJK develops from multifactorial etiologies and teriparatide was prescribed based on individual physician discretion rather than standardized criteria, we employed propensity score matching (PSM) to control for potential selection bias and confounding variables. Variables demonstrating statistically significant differences between the teriparatide and non-teriparatide groups in the unmatched cohort were included in the matching algorithm. These variables included age, preoperative PI-LL, preoperative TPA, UIV cementing, UIV screw angle, and fusion length. A logistic regression model was used to estimate the propensity score, representing the probability of receiving teriparatide treatment given the observed covariates. A 1:2 nearest-neighbor matching algorithm was employed with a caliper width of 0.2 standard deviations (SD) of the logit of the propensity score. Standardized mean differences (SMD) were calculated for all baseline variables, with SMD < 0.1 and between 0.1 and 0.2 indicating excellent and acceptable balance, respectively, between the matched groups.

**Statistical Analysis:** Continuous variables are presented as means ± SD and were compared using Student’s *t*-tests or Mann–Whitney U tests, as appropriate. Categorical variables are expressed as frequencies (percentages) and were compared using chi-square tests or Fisher’s exact tests. Univariate and multivariate logistic regression analyses were performed to assess the association between teriparatide use and PJK outcomes and to identify independent risk factors for PJK development. Variables with *p* < 0.1 in univariate analysis were entered into the multivariate model. Results are presented as odds ratios (ORs) with 95% confidence intervals (CIs). Statistical analyses were performed using Python (version 3.10). A *p*-value of <0.05 was considered statistically significant.

## 3. Results

### 3.1. Baseline Characteristics Before Matching

A total of 292 patients met the inclusion criteria: 59 in the teriparatide group and 233 in the non-teriparatide group. Baseline demographic and clinical characteristics are summarized in Table 1. The teriparatide group was significantly older (72.3 vs. 70.5 years, *p* = 0.047) and exhibited more severe sagittal deformity, as indicated by a greater preoperative PI-LL mismatch (45.4° vs. 38.9°, *p* = 0.028) and a higher preoperative TPA (36.4° vs. 31.9°, *p* = 0.014). Additionally, the teriparatide group had higher rates of UIV cementing (59.3% vs. 26.6%, *p* < 0.001), more caudal UIV screw angle (0.8° vs. 2.6°, *p* = 0.005), and longer fusion length (7.0 vs. 6.3 levels, *p* = 0.007).

### 3.2. Matched Cohort Characteristics

**Figure 1:**
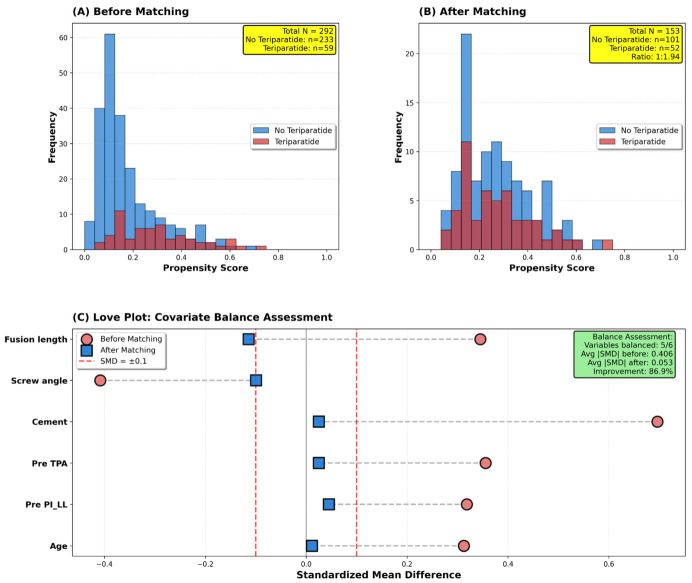
Assessment of propensity score matching: (**A**) distribution of propensity scores before matching, (**B**) distribution of propensity scores after matching, and (**C**) Love plot illustrating changes in standardized mean difference (SMD) for six covariates before and after matching. The gray dotted lines connect the pre- and post-matching SMD values for each covariate.

**Figure 2:**
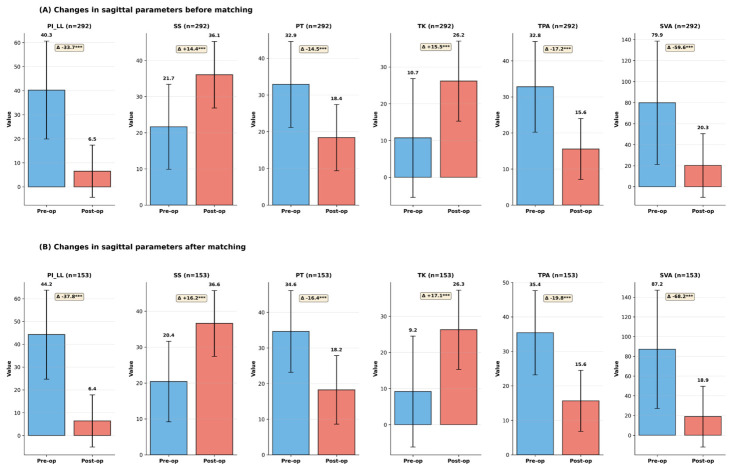
Perioperative changes in sagittal parameters: (**A**) before matching and (**B**) after matching. *** *p* < 0.001.

PSM was performed using six covariates: age, preoperative PI-LL, preoperative TPA, UIV cementing, UIV screw angle, and fusion length. After matching, 153 patients remained (52 in the teriparatide group and 101 in the non-teriparatide group), resulting in a matching ratio of 1:1.94 (Figure 1A,B). Covariate balance was achieved for 5 of 6 variables, with all SMDs below 0.1, except for fusion length (SMD = 0.114) (Figure 1C). The average absolute SMD improved from 0.406 before matching to 0.053 after matching, representing an 86.9% reduction (Figure 1C). For all 31 included variables, no significant differences were observed in demographic data, preoperative radiographic parameters, surgical variables, or preoperative and postoperative radiographic parameters (all *p* values > 0.05) (Table 2). Among these, 23 variables exhibited excellent balance (SMD < 0.1), while 8 variables showed acceptable balance (SMD between 0.1 and 0.2). Postoperatively, all sagittal parameters demonstrated significant changes, including PI-LL, SS, PT, TK, TPA, and SVA. The patterns of change were similar before matching (Figure 2A) and after matching (Figure 2B).

### 3.3. PJK and Clinical Outcomes

**Figure 3:**
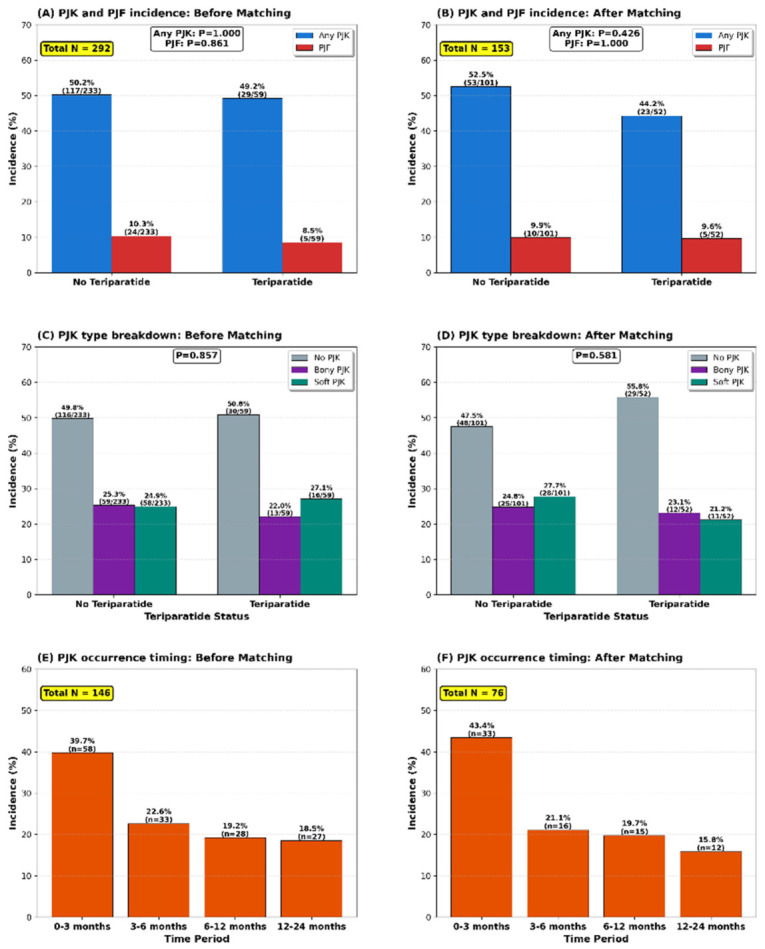
PJK outcomes: (**A**) incidence of PJK and PJF before matching, (**B**) incidence of PJK and PJF after matching, (**C**) types of PJK before matching, (**D**) types of PJK after matching, (**E**) time to develop PJK before matching, and (**F**) time to develop PJK after matching.

Before matching, the incidence of any PJK was similar between groups (50.2% vs. 49.2%, *p* = 1.000), as was the incidence of PJF (10.3% vs. 8.5%, *p* = 0.861) (Figure 3A). After matching, the incidence of PJK did not differ significantly between the groups (52.5% vs. 44.2%, *p* = 0.426). Similarly, PJF incidence was comparable between groups (9.9% vs. 9.6%, *p* = 1.000) (Figure 3B). The distribution of PJK types before matching did not differ significantly between the non-teriparatide and teriparatide groups (*p* = 0.857) (Figure 3C); bony PJK occurred in 25.3% of the non-teriparatide group and 22.0% of the teriparatide group, while soft PJK occurred in 24.9% and 27.1%, respectively. After matching, the distribution of PJK types remained similar between groups (*p* = 0.581) (Figure 3D); bony PJK occurred in 24.8% versus 23.1%, and soft PJK in 27.7% versus 21.2%, respectively.

Among the 146 patients who developed PJK before matching, most cases occurred within the first 3 months postoperatively (39.7%), followed by 3–6 months (22.6%), 6–12 months (19.2%), and 12–24 months (18.5%) (Figure 3E). In the matched cohort, 76 patients developed PJK with a similar temporal distribution (Figure 3F); the highest incidence occurred within 0–3 months (43.4%), followed by 3–6 months (21.1%), 6–12 months (19.7%), and 12–24 months (15.8%). Among all patients in the teriparatide group, the mean treatment duration was 4.9 months before surgery and 3.7 months after surgery, for a total of 8.6 months (Table 3). The durations of teriparatide use, whether before surgery (*p* = 0.653), after surgery (*p* = 0.847), or the total duration (*p* = 0.604), did not differ significantly among PJK types.

In the matched cohort, no significant differences were found in clinical outcome measures, including ODI, SRS-22r, and SF-36 PCS, either preoperatively or at the 2-year follow-up, between the two groups (Table 4).

### 3.4. Risk Factors for PJK

Univariate logistic regression analysis identified several factors associated with PJK development in the unmatched overall cohort, including older age, higher ASA grade, adverse preoperative spinopelvic parameters (PI-LL, SS, PT, and TPA), surgical factors (caudal UIV screw angle and UIV at T11-L1), and postoperative sagittal alignment (PI-LL, SS, and TK) (Table 5). Teriparatide use was not significantly associated with PJK in the univariate analysis (OR = 0.958, *p* = 0.884).

Multivariate logistic regression analysis identified older age (OR = 1.058, *p* = 0.018), higher ASA grade (OR = 2.603, *p* = 0.002), UIV at T11-L1 (OR = 2.786, *p* = 0.005), and greater postoperative TK (OR = 1.044, *p* = 0.003) as independent risk factors for PJK. After adjusting for confounders, teriparatide use was not an independent predictor of PJK (OR = 0.872, *p* = 0.684).

## 4. Discussion

Teriparatide exerts anabolic effects on bone metabolism by stimulating osteoblast activity and promoting new bone formation. Previous studies have demonstrated significant improvements in bone quality parameters following perioperative administration. Yagi et al. reported that the perioperative use of teriparatide significantly improved volumetric bone mineral density and fine bone structure at the UIV + 1 vertebra, resulting in reduced bone failure-type PJK in patients with ASD [16]. Similarly, Maruo et al. found that Hounsfield units at the UIV increased significantly more in the teriparatide group than in the non-teriparatide group (20.8% vs. 10.2%, *p* < 0.001) [22]. These findings provide a strong rationale for expecting teriparatide to reduce the development of PJK, particularly bony PJK, through improved bone quality at the proximal junction where biomechanical stress is concentrated. Despite this rationale, our study found that preoperative teriparatide did not significantly reduce the incidence of PJK or PJF in patients with compromised bone quality (T-score < −1.0) undergoing long-segment fusion for ASD. After propensity score matching, PJK incidence was 44.2% in the teriparatide group versus 52.5% in the controls (*p* = 0.426), and PJF rates were comparable (9.6% vs. 9.9%, *p* = 1.000).

The role of teriparatide in preventing PJK remains controversial in recent literature. Although teriparatide has shown promising results, the current evidence supporting its role in preventing proximal junctional complications remains limited, as most available studies are retrospective with relatively small sample sizes and high-quality randomized evidence remains scarce. While some early studies reported beneficial effects [15,16], recent studies with more robust designs have yielded mixed results regarding its preventive efficacy against PJK. In a randomized controlled trial comparing perioperative teriparatide with denosumab in osteoporotic patients, the reduction in PJK was not statistically significant, while PJF was significantly lower in the teriparatide group [18]. Mohanty et al. conducted a propensity score-matched analysis in ASD surgery and demonstrated that while teriparatide reduced overall complication rates in osteoporotic patients undergoing long spinal fusion, it did not specifically decrease the incidence of proximal junctional complications [14]. Furthermore, a systematic review by Echt et al. concluded that perioperative teriparatide therapy, despite representing the strongest pharmacological evidence available, still showed only level III evidence for PJK prevention, with significant heterogeneity across studies [17]. This limited and heterogeneous evidence may partly explain the absence of a clear consensus regarding the routine use of preoperative teriparatide as a surgical adjuvant. The convergence of these findings suggests a more complex relationship between bone quality improvement and PJK prevention than initially hypothesized, indicating that local bone density enhancement alone may be insufficient to prevent PJK in the multifactorial context of ASD surgery.

Multivariate analysis in this study identified several independent risk factors for PJK, including older age, higher ASA grade, thoracolumbar junction UIV placement, and greater postoperative TK, which aligns with previous literature [4,5,23,24]. Bone-related parameters, including T-score and UIV Hounsfield units, were not identified as independent risk factors in our multivariate analysis. This suggests that bone quality alone may exert a relatively limited influence on proximal junctional complications compared with biomechanical and patient-related factors. Notably, age and ASA grade are non-modifiable patient characteristics that cannot be altered through surgical planning. In contrast, UIV selection and postoperative sagittal alignment are modifiable surgical factors that require particular attention in patients with poor bone quality. The thoracolumbar junction serves as a transition zone between the rigid thoracic spine and the mobile lumbar spine, making it susceptible to increased mechanical stress. Greater postoperative TK further compounds this biomechanical disadvantage by increasing the sagittal moment arm and lever forces at the proximal junction. Therefore, in patients with compromised bone quality, careful consideration of modifiable surgical factors—particularly avoiding UIV placement at the thoracolumbar junction when feasible and achieving appropriate sagittal alignment—may be more critical for PJK prevention than enhancing bone quality alone.

Regarding treatment duration, our cohort received teriparatide for a mean of 8.6 months (4.9 months preoperatively and 3.7 months postoperatively), which is shorter than the previous protocols reported by Yagi et al. (18 months postoperatively) [16] and Mohanty et al. (24 months total) [14]. However, the relatively short postoperative treatment duration is unlikely to have significantly influenced PJK prevention, given that two-thirds of PJK cases in our series occurred within the first 6 months postoperatively. Maruo et al. reported that patients who developed PJF had significantly shorter preoperative teriparatide treatment compared to those without PJF (34.7 vs. 86.9 days, *p* = 0.004) [22]. In our study, all patients in the teriparatide group received at least 3 months of preoperative treatment (mean 4.9 months), which exceeds the threshold associated with PJF risk in Maruo’s study. Because teriparatide is generally administered for a limited cumulative duration, its use as a perioperative surgical adjuvant should be balanced against the patient’s long-term osteoporosis management strategy; this issue was not directly evaluated in the present study.

Several limitations should be acknowledged. The single-center retrospective design and non-randomized allocation of patients introduce potential selection bias, despite the use of propensity score matching. The initiation and termination of teriparatide were based on physician discretion rather than standardized criteria, which may have led to heterogeneous treatment indications. Although available medication records were reviewed, complete information on concomitant anti-osteoporotic medications, such as bisphosphonates or denosumab, could not be reliably ascertained because of the retrospective design, particularly for prescriptions from outside institutions and remote medication histories. Therefore, residual confounding related to concomitant anti-osteoporotic treatment may have affected between-group differences in bone quality and attenuated the observed treatment effect of teriparatide. We did not obtain repeat DEXA scans or measure Hounsfield units after treatment, preventing a direct assessment of bone quality improvement. The relatively short duration of postoperative treatment may have limited its protective effects. Finally, our study specifically focused on patients with a T-score < −1.0, which may limit the generalizability of the results to patients with osteoporosis or normal bone density.

## 5. Conclusions

In patients with compromised bone quality undergoing long-segment instrumented fusion for ASD, preoperative teriparatide did not significantly reduce the incidence of PJK or PJF. While teriparatide may enhance local bone density, improvement in bone quality alone appears insufficient to prevent PJK due to the complex biomechanical and patient-related factors inherent in extensive deformity correction.

## Figures and Tables

**Table 1:** Comparison of baseline data before matching.

Variables	No Teriparatide (*N* = 233)	Teriparatide (*N* = 59)	*p*
Sex (female), *n* (%)	213 (91.4%)	58 (98.3%)	0.067
Age (years)	70.5 ± 6.5	72.3 ± 5.0	**0.047**
BMI (kg/m^2^)	25.6 ± 3.5	25.5 ± 3.0	0.760
ASA grade	2.2 ± 0.6	2.2 ± 0.4	0.823
FI-5 score	1.3 ± 0.9	1.5 ± 0.9	0.082
T-score	−2.0 ± 0.6	−2.2 ± 0.8	0.070
Hounsfield unit at the UIV	102.8 ± 34.3	98.3 ± 35.9	0.370
Preoperative PI-LL (°)	38.9 ± 20.1	45.4 ± 20.7	**0.028**
Preoperative SS (°)	22.2 ± 11.6	19.5 ± 12.2	0.112
Preoperative PT (°)	33.6 ± 11.3	35.8 ± 12.6	0.071
Preoperative TK (°)	10.8 ± 15.8	10.5 ± 17.7	0.896
Preoperative TPA (°)	31.9 ± 12.4	36.4 ± 12.8	**0.014**
Preoperative SVA (mm)	77.8 ± 58.8	88.2 ± 57.8	0.223
Prior lumbar fusion, *n* (%)	85 (36.5%)	24 (40.7%)	0.552
LLIF, *n* (%)	186 (79.8%)	48 (81.4%)	0.793
Pelvic fixation, *n* (%)	187 (80.3%)	46 (78.0%)	0.695
UIV cementing, *n* (%)	62 (26.6%)	35 (59.3%)	**<0.001**
UIV screw angle (°)	2.6 ± 4.4	0.8 ± 4.7	**0.005**
Fusion length (levels)	6.3 ± 2.2	7.0 ± 1.8	**0.007**

Bold *p*-values indicate statistical significance. A positive UIV screw angle indicates a cranial direction relative to the upper endplate.

**Table 2:** Comparison of variables after 1:2 matching.

Variables	No Teriparatide (*N* = 101)	Teriparatide (*N* = 52)	*p*	SMD
**Demographic data**				
Sex (female), *n* (%)	96 (95.0%)	51 (98.1%)	0.361	0.038
Age (years)	71.9 ± 5.6	72.0 ± 4.9	0.949	0.011
BMI (kg/m^2^)	25.7 ± 3.6	25.7 ± 3.1	0.908	0.020
ASA grade	2.2 ± 0.5	2.1 ± 0.4	0.573	0.046
FI-5 score	1.2 ± 0.8	1.4 ± 0.9	0.113	0.129
T-score	−2.1 ± 0.6	−2.2 ± 0.8	0.354	0.159
Hounsfield unit at the UIV	102.6 ± 31.8	98.0 ± 36.3	0.424	0.137
**Preoperative radiographic data**				
Preoperative PI-LL (°)	43.9 ± 19.6	44.8 ± 19.5	0.793	0.045
Preoperative SS (°)	20.5 ± 11.6	20.2 ± 10.6	0.856	0.031
Preoperative PT (°)	34.4 ± 11.4	35.0 ± 11.8	0.751	0.054
Preoperative TK (°)	8.4 ± 15.6	10.7 ± 14.9	0.393	0.146
Preoperative TPA (°)	35.3 ± 12.2	35.6 ± 12.5	0.883	0.025
Preoperative SVA (mm)	86.9 ± 59.7	87.5 ± 61.2	0.958	0.009
**Surgical data**				
Prior lumbar fusion, *n* (%)	32 (31.7%)	20 (38.5%)	0.402	0.053
LLIF, *n* (%)	83 (82.2%)	43 (82.7%)	0.937	0.001
Pelvic fixation, *n* (%)	84 (83.2%)	42 (80.8%)	0.712	0.012
UIV cementing, *n* (%)	57 (56.4%)	30 (57.7%)	0.882	0.025
UIV screw angle (°)	1.8 ± 4.1	1.4 ± 4.6	0.560	0.099
Fusion length (levels)	7.0 ± 2.1	6.8 ± 1.8	0.514	0.115
UIV levels			0.506	0.094
L2, *n* (%)	19 (18.9%)	14 (26.9%)		
T11-L1, *n* (%)	25 (24.8%)	11 (21.2%)		
≥T10, *n* (%)	57 (56.4%)	27 (51.9%)		
**Postoperative radiographic data**				
Postoperative PI-LL (°)	6.5 ± 12.3	6.2 ± 9.5	0.862	0.030
Postoperative SS (°)	36.6 ± 9.4	36.7 ± 8.8	0.945	0.012
Postoperative PT (°)	17.9 ± 10.1	18.8 ± 8.7	0.581	0.094
Postoperative TK (°)	26.2 ± 11.5	26.5 ± 9.9	0.874	0.027
Postoperative TPA (°)	15.4 ± 9.0	16.1 ± 8.6	0.668	0.073
Postoperative SVA (mm)	20.0 ± 31.3	16.8 ± 29.9	0.539	0.105
GAP score	4.3 ± 3.2	4.7 ± 2.9	0.421	0.138
GAP category			0.328	0.121
Proportioned, *n* (%)	33 (32.7%)	11 (21.2%)		
Moderately disproportioned, *n* (%)	46 (45.5%)	28 (53.8%)		
Severely disproportioned, *n* (%)	22 (21.8%)	13 (25.0%)		
SAAS	2.4 ± 2.9	2.3 ± 2.5	0.759	0.025
SAAS category			0.481	0.098
Undercorrection, *n* (%)	13 (12.9%)	4 (7.7%)		
Matched correction, *n* (%)	19 (18.8%)	13 (25.0%)		
Overcorrection, *n* (%)	69 (68.3%)	35 (67.3%)		
Roussouly classification			0.838	0.002
Restored, *n* (%)	41 (40.6%)	22 (42.3%)		
Non-restored, *n* (%)	60 (59.4%)	30 (57.7%)		

Bold *p*-values indicate statistical significance. A positive UIV screw angle indicates a cranial direction relative to the upper endplate. SMDs are presented as absolute values. SMD, standardized mean difference.

**Table 3:** Duration of teriparatide treatment in the teriparatide group.

Duration	Overall Cohort (*N* = 52)	No PJK (*N* = 29)	Bony PJK (*N* = 12)	Soft PJK (*N* = 11)	*p*
Before surgery (months)	4.9 ± 2.3	4.7 ± 2.1	4.8 ± 2.4	5.5 ± 2.7	0.653
After surgery (months)	3.7 ± 2.6	3.5 ± 2.4	3.9 ± 2.8	3.9 ± 3.1	0.847
Total duration (months)	8.6 ± 3.2	8.2 ± 2.8	8.7 ± 3.9	9.4 ± 3.7	0.604

**Table 4:** Comparison of clinical outcomes between the groups in the matched cohort.

	No Teriparatide (*N* = 101)	Teriparatide (*N* = 52)	*p*
Preoperative ODI	62.6 ± 16.5	61.6 ± 14.1	0.727
Preoperative SRS-22r	2.2 ± 0.5	2.3 ± 0.5	0.782
Preoperative SF-36 PCS	24.2 ± 13.7	23.4 ± 16.3	0.720
2-year ODI	37.0 ± 17.7	32.3 ± 21.0	0.190
2-year SRS-22r	3.4 ± 0.8	3.5 ± 0.8	0.497
2-year SF-36 PCS	52.3 ± 24.8	57.0 ± 27.6	0.433

ODI, Oswestry Disability Index; SRS-22r, Scoliosis Research Society-22r questionnaire; SF-36, Short Form-36 questionnaire; PCS, Physical Component Score.

**Table 5:** Logistic regression analysis for risk factors of PJK in the unmatched overall cohort.

Variables	Univariate Analysis		Multivariate Analysis		
	OR	*p*	OR	95% CI	*p*
Teriparatide use	0.958	0.884	0.872	0.450–1.688	0.684
Female (vs. male)	1.108	0.821			
Age (years)	1.060	**0.003**	1.058	1.010–1.109	**0.018**
BMI (kg/m^2^)	1.042	0.234			
ASA grade	2.690	**<0.001**	2.603	1.410–4.808	**0.002**
FI-5 score	1.148	0.281			
T-score	0.696	0.863			
Hounsfield unit at the UIV	1.005	0.164			
Preoperative PI-LL (°)	1.013	**0.034**	1.024	0.994–1.055	0.118
Preoperative SS (°)	0.968	**0.002**	0.981	0.945–1.018	0.314
Preoperative PT (°)	1.024	**0.024**	1.005	0.959–1.053	0.851
Preoperative TK (°)	0.997	0.652			
Preoperative TPA (°)	1.021	**0.032**	0.989	0.939–1.041	0.674
Preoperative SVA (mm)	1.003	0.117			
Prior lumbar fusion	1.381	0.184			
LLIF	1.090	0.769			
Pelvic fixation	1.469	0.191			
UIV cementing	1.242	0.385			
UIV screw angle (°)	1.056	**0.041**	1.044	0.983–1.109	0.158
Fusion length (levels)	1.062	0.280			
UIV levels		**0.002**			**0.002**
L2	Ref.	-	Ref.	-	-
T11-L1	3.133	**0.001**	2.786	1.369–5.670	**0.005**
≥T10	1.870	**0.028**	1.259	0.647–2.449	0.497
Postoperative PI-LL (°)	0.991	0.383			
Postoperative SS (°)	0.974	**0.046**	0.981	0.945–1.020	0.334
Postoperative PT (°)	1.017	0.203			
Postoperative TK (°)	1.029	**0.010**	1.044	1.015–1.074	**0.003**
Postoperative TPA (°)	1.020	0.165			
Postoperative SVA (mm)	1.001	0.853			
GAP score	1.056	0.161			
GAP category		0.293			
Proportioned	Ref.				
Moderately disproportioned	0.890	0.676			
Severely disproportioned	1.414	0.294			
SAAS	1.024	0.589			
SAAS category		0.373			
Undercorrection	1.681	0.229			
Matched correction	Ref.				
Overcorrection	1.373	0.240			
Roussouly restored (vs. non-restored)	0.734	0.193			

Bold *p*-values indicate statistical significance. A positive UIV screw angle indicates a cranial direction relative to the upper endplate.

## Data Availability

Data are not publicly available due to patient privacy and institutional regulation but are available from the corresponding author upon reasonable request.

## Abbreviations

The following abbreviations are used in this manuscript:

ASD	Adult spinal deformity
DEXA	Dual-energy X-ray absorptiometry
FI-5	5-item modified frailty index
ODI	Oswestry Disability Index
PI-LL	Pelvic incidence minus lumbar lordosis
PJF	Proximal junctional failure
PJK	Proximal junctional kyphosis
PSM	Propensity score matching
SF-36 PCS	Short Form-36 Physical Component Score
SRS-22r	Scoliosis Research Society-22r questionnaire
SVA	Sagittal vertical axis
TK	Thoracic kyphosis
TPA	T1 pelvic angle
UIV	Upper instrumented vertebra
